# Free Drugs and State Control

**Published:** 1916-08-19

**Authors:** 


### The Hospital

The Workers' Newspaper of Administrative Medicine and Institutional Life, Administration, National Insurance and Health.

No. 1576, Vol. LX. SATURDAY, AUGUST 19, 1916. PRICE ONE PENNY

### FREE DRUGS AND STATE CONTROL.

One of the recommendations of a recent Royal Commission is to the effect that salvarsan and other expensive drugs shall be supplied gratuitously to medical practitioners, and following on this, almost inevitably, is the suggestion that applicants for this privilege shall show that they have some special competence to administer the remedies thus obtained. The question therefore arises where the judgment as to competence is to be placed, and the only practical proposal which has so far appeared is that the decision is to rest with the medical officer in charge of the local clinic devoted to the treatment of the disease for which the remedies in question are intended. At first sight this may appear to be a mere matter of administration, but a closer examination shows that a very serious question of principle is involved. Hitherto a medical practitioner who has obtained a legal qualification to practise has had the full right to undertake any professional procedure which he desires, this liberty being, of course, associated with the qualification that he accepts full responsibility for any treatment which he puts into operation. Naturally a man of prudence and common sense takes steps to equip himself both with information and, where it is necessary, with technical skill before entering upon methods which to him at least are novel and unaccustomed. But this responsibility has been a moral claim and not a formal and technical bond. If the new proposal is to come into operation there will be a fresh condition in reference at least to a certain branch of practice. In each neighbourhood there will be one group of practitioners who will be authoritatively labelled as competent to administer salvarsan and its allies, and another group who will lack this official distinction. Further, the conditions which will entitle any practitioner to claim the more positive position are almost certain to vary in different districts, for there is no force to ensure uniformity of method and of decision among the various medical officers with whom the verdict will rest. In other words, for men already legally qualified a test is to be erected in reference to their capacity to adopt a certain form of treatment, and the standard of the test is so far left to the decision of individual caprice. The proposal, we repeat, is a very serious one, and it will be well for the medical profession to scrutinise it carefully and even jealously. At present the profession enjoys liberty in its election of judgment and method, the liberty being tempered by individual responsibility. Now appears a new standard, at least in a certain limited sphere, and if this is accepted the freedom hitherto enjoyed is in a corresponding measure invaded. Moreover, there is the force of precedent to be considered, and the limited regulation now proposed may easily become a justification for further measures of the same order.

It is hardly necessary for us to say that we are no advocates for the adoption by practitioners of methods of treatment for which they have not adequately prepared themselves. Nor do we believe that the great body of the profession are in need of any special warning on this point. There is abundant evidence that practitioners generally are ready to seek a practical knowledge of new developments before they proceed to practise them, and that failing the opportunity or inclination for such experience they prefer to transfer the patient to some more fully equipped colleague. In the present instance nothing but blame would be due to the man who, without the most careful study and preparation, would propose to inject into the blood-stream of a patient such powerful drugs as are now in question. To treat such a proceeding lightly and as one of trivial responsibility is, we believe, wholly alien to the spirit and practice of the modern practitioner. If such a one there be we are convinced he is an exception, and censure and not support will he receive from the public opinion of the profession. This high standard of responsibility is a well-established tradition, and in these columns certainly nothing will ever be said which is calculated to weaken it. It rests in some sense upon individual judgment, but this judgment is educated by habit and by the sense of a general professional verdict which is sufficiently penetrating and sufficiently emphatic to hinder rash and unjustifiable enterprises. The practitioner is free to elect any course for which he regards himself as capable, but he must be ready for the judgment of his own conscience and for the standard of his *confrères*. The present proposal is an invasion of this position, inasmuch as it imposes an outside judgment and decrees for the already qualified man a new set of tests to which he must fain conform if he is to boast the official ticket and to enjoy the privileges granted to certain of his colleagues. The verdict is no longer with himself but with an outside tribunal. This is a new principle in the general atmosphere of the profession, and if it is accepted it may have far-reaching consequences.

If, however, the proposal is looked at from the official point of view a strong case may be argued in its favour. The State, it can be claimed, is willing for its own ends to supply to practitioners certain expensive remedies, and it therefore has the right to know that the recipients are qualified to use such remedies with advantage. For ourselves we do not see how this argument can be challenged. Still more, how can the State be resisted if it demands, as it is morally certain to do, to be informed in what circumstances the gratuitous remedies have been employed and with what effect? If the profession accepts public subsidies it must in corresponding measure submit to public control. Hence, for those who believe that both public and professional advantage are attached to the independence and personal responsibility of private medical practice there is no way open but the refusal of the offered subsidies. In the current phrase of the hour, the profession cannot have it both ways. If they accept State help they must be ready to answer State-framed questions. If they object to these questions they must deny the preliminary gifts. No doubt it is tempting to hear of free supplies, but let it be recognised that such an arrangement carries consequences in the shape of official interference. For those who wish to keep clear of such interference there is only one safe course, and this is to decline official help. The Greeks bearing gifts have a reputation which rests on many experiences.

